# The association between infant sleep, cognitive, and psychomotor development: a systematic review

**DOI:** 10.1093/sleep/zsae174

**Published:** 2024-09-04

**Authors:** Bryan Butler, Rebecca Burdayron, Gil Mazor Goder, Clara Lewis, Mélanie Vendette, Bassam Khoury, Marie-Hélène Pennestri

**Affiliations:** Department of Educational and Counselling Psychology, McGill University, Montréal, QC, Canada; Hôpital en Santé Mentale Rivière-des-Prairies, CIUSSS-du-Nord-de-l’île-de-Montréal, Montréal, QC, Canada; Department of Educational and Counselling Psychology, McGill University, Montréal, QC, Canada; Hôpital en Santé Mentale Rivière-des-Prairies, CIUSSS-du-Nord-de-l’île-de-Montréal, Montréal, QC, Canada; Department of Educational and Counselling Psychology, McGill University, Montréal, QC, Canada; Department of Educational and Counselling Psychology, McGill University, Montréal, QC, Canada; Center for Advanced Research in Sleep Medicine, Hôpital du Sacré-Coeur de Montréal, CIUSSS du Nord-de-l’Ile-de-Montréal, Montréal, QC, Canada; Department of Educational and Counselling Psychology, McGill University, Montréal, QC, Canada; Department of Educational and Counselling Psychology, McGill University, Montréal, QC, Canada; Hôpital en Santé Mentale Rivière-des-Prairies, CIUSSS-du-Nord-de-l’île-de-Montréal, Montréal, QC, Canada

**Keywords:** pediatrics, infants, cognitive development, sleep/wake cognition

## Abstract

**Study Objectives:**

To synthesize findings of original articles examining the association between sleep–wake patterns of typically developing infants aged 0 to 18 months and cognitive and psychomotor development.

**Methods:**

A systematic search strategy was used to identify articles assessing the association between infant sleep (0 to 18 months) and cognitive/psychomotor development (Medline, PsycINFO, and SCOPUS). Of 7136 articles screened, 22 articles met inclusion criteria, and the results were subsequently synthesized. A quality assessment was conducted, and studies were categorized as “poor,” “fair,” or “good.”

**Results:**

Out of 22 studies, 2 found exclusively significant associations (SAs) between infant sleep and cognitive/psychomotor development, 2 found no SAs and 17 found mixed results (MRs). Studies with exclusively significant results used a single sleep variable and single timepoint designs. Studies finding MRs or no SAs used multiple sleep, developmental variables, or multi-timepoint designs. Eight out of 10 studies and 7 out of 8 studies investigating nocturnal and total sleep duration, respectively, found no SA with developmental outcomes. While 63% of studies were rated as having good methodological quality, all studies but one had an estimated power of less than 0.80.

**Conclusions:**

Findings of this review do not support conclusive associations between sleep–wake patterns in infancy and cognitive/psychomotor development. This conclusion contrasts with the literature in older populations, questioning if the association between sleep and development is of a different nature in infancy, potentially because of brain maturation. More studies including larger samples will be needed to clarify the presence or absence of such an association.

Sleep is a key factor involved in child development and brain maturation [1–5]. The association between sleep and cognitive functioning has been well investigated in adults and in children [6–8]. For instance, shorter sleep duration has been associated with lower cognitive performance in preschool and school-aged children [6, 9, 10]. Sleep problems in early childhood have also been associated with poorer school achievement scores [11, 12].

While the association between sleep and cognitive functioning has been explored further in adults and children, less is known about this association in infancy. Indeed, sleep is a developmental process, and sleep–wake patterns in infancy are markedly different than those in older children [9, 11, 13–16]. During the first several weeks of life, infants spend approximately 16–18 hours/day in a sleeping state [17–19]. During this period, sleep is highly fragmented with infants sleeping for short periods of 2–3 hours [17, 20, 21]. Moreover, infants’ ability to differentiate between day and night during early infancy is limited as their circadian rhythm is in a developmental state [16]. Sleep stages during early infancy differ from those in older populations. During infancy, rapid eye movement (REM) and non-REM sleep are classified as quiet sleep (QS), active sleep (AS), and indeterminate (IS) sleep, until approximately 2 months of age [22, 23].

Gradually, the percentage of nocturnal sleep will increase over time, while nocturnal awakenings will decrease as well as the number and duration of daytime naps [24]. The development and consolidation of the sleep–wake cycle in infancy is influenced by multiple internal (e.g., circadian timing system and temperament) and external (e.g., light exposure and parental behaviors) factors [16, 25–30]. One of the main challenges related to the investigation of sleep during infancy is that it is a developmental process, whose maturation varies significantly from infant to infant [15, 17, 20]. Moreover, major variations also occur from night to night within the same infant [31, 32]. Considering the inter- and intra-individual variability and the developmental aspect, it is unclear whether infant sleep patterns are associated with measures of cognitive and psychomotor functioning in early development. The associations observed in older children and adults may not be applicable during earlier developmental periods.

To that effect, a previous review conducted by Ednick et al. [33] in 2009 examined the association between sleep and development during the first year of life. Based on the 18 studies included in the review, the authors concluded that a causal relationship could not be drawn concerning infant sleep and its association with cognitive and psychomotor development during this period of development. Moreover, the authors observed that findings from one developmental period (e.g., first 48 hours of life; 6 months) could not be generalized to another, emphasizing the developmental nature of sleep during infancy. While this review paper clearly expanded the understanding of the association between infant sleep and development during early infancy, it did not employ a systematic search process nor include a quality assessment. Moreover, both full-term and preterm infants were included in the review, introducing an important confounding variable, since sleep in preterm infants exhibits distinct characteristics and developmental trajectories [20, 34, 35]. Likewise, the methodology was also not explicit regarding the inclusion or exclusion of other comorbidities, such as neurological, physiological, or genetic disorders. Finally, new studies may have been published since Ednick et al.’s review [33] and could help to refine and update our understanding of the association between infant sleep and developmental outcomes.

Building on this previous review, the present systematic review focuses on the associations between infant sleep and cognitive and psychomotor development in full-term healthy infants aged 0–18 months. Specifically, the aims of this review are to (1) synthesize the available research assessing the association between infant sleep (<18 months) and cognitive and psychomotor development in typically developing infants and (2) assess and critique the current quality of knowledge. This systematic review will provide updated knowledge and offer an overview of the current available research. Given the developmental nature of infant sleep, results will be organized and presented as a function of age.

## Methods

This systematic review was registered with the International Prospective Register of Systematic Reviews (CRD42019123272) and conducted according to the Preferred Reporting Items for Systematic Reviews and Meta-Analyses (PRISMA) [36].

### Article search and selection

The search strategy was designed by the research team in collaboration with a research librarian. All databases were searched on August 17, 2018, and included MEDLINE (1946–Present), PsycINFO (1806–Present), and SCOPUS. The keyword *Sleep* was solely used while searching MEDLINE and PsycINFO, as the addition of *Cognitive* and *Psychomotor* produced limited results. The search strategy can be accessed in Supplementary Appendix A. All results were imported into EndNote and duplicate records were discarded using the de-duplicate method developed by Bramer et al. [37]. Updated searches were conducted on February 14, 2020, September 7, 2022, and March 10, 2024, and yielded eight additional studies, which met our inclusion criteria.

**Figure 1.**
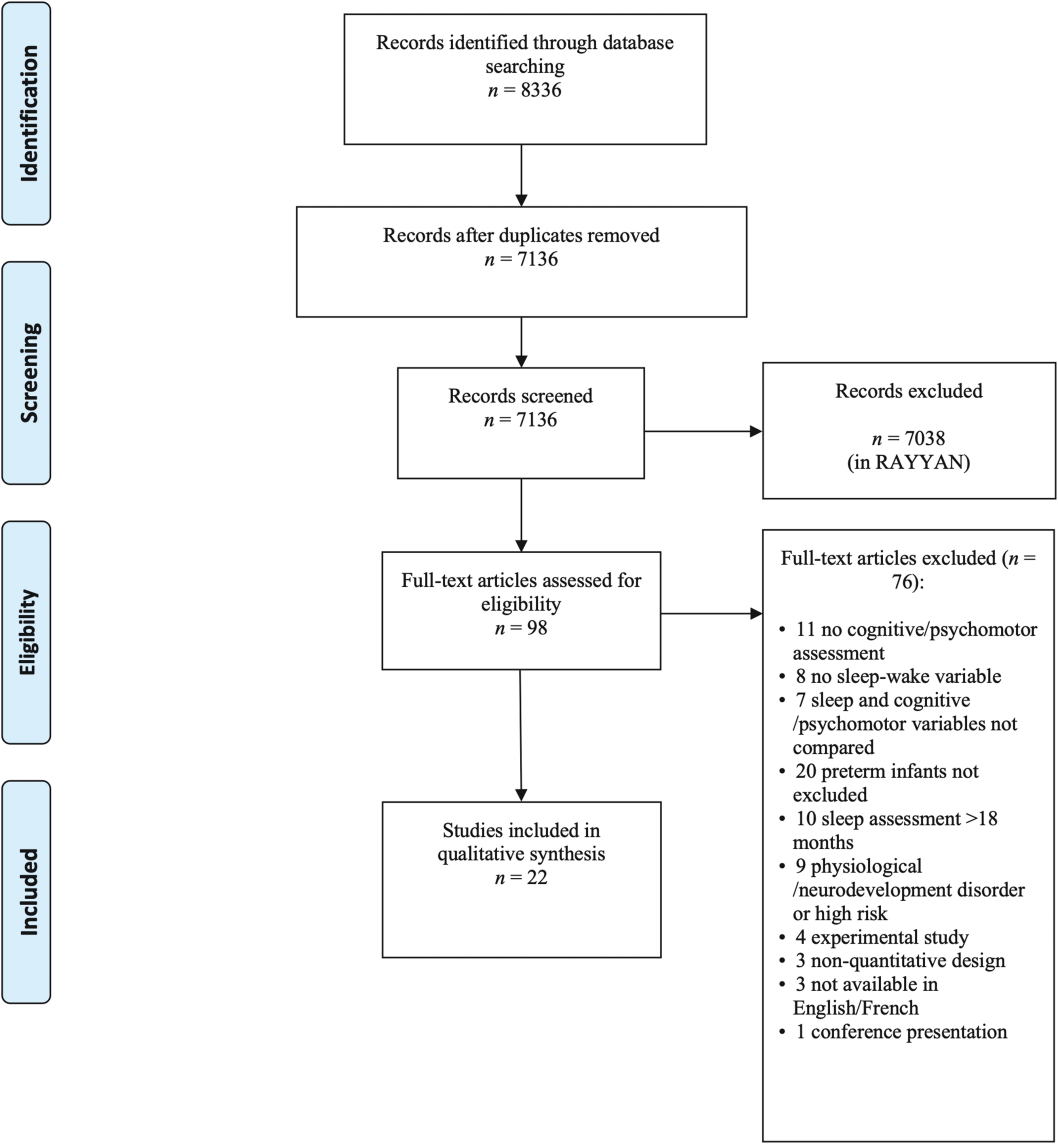
PRISMA flowchart.

The subsequent stages of the review were conducted independently by two researchers (B.B. and G.M.-G. or R.B.) to minimize bias. A third member of the research team was available to consult in the event of a disagreement. The search yielded a total of 8336 articles. After the removal of duplicates, 7136 abstracts were screened in Rayyan—a web and mobile app for systematic reviews [38] for the following inclusion criteria: (1) ≤18 months of age at time of sleep assessment, (2) born at full-term (≥37 weeks), (3) absence of neurological, physiological, and/or genetic disorders (autism spectrum disorder Down’s syndrome, epilepsy, asthma, etc.), (4) assessed participant sleep, either with an objective or self-reported measure (e.g. polysomnography, actigraphy, observation, time-lapse video, motility monitoring system, sleep diary, sleep questionnaire, etc.), (5) assessed participant cognitive and/or psychomotor functioning (e.g. Bayley Scales of Infant Development, Ages and Stages Questionnaire, Wechsler Preschool & Primary Scale of Intelligence, etc.), (6) full-text available in English or French, (7) only empirical quantitative studies were included (i.e., case studies and review articles were excluded), and (8) experimental studies in which diurnal sleep (napping) was manipulated to assess its immediate impact on a specific cognitive test. Based on these criteria, the full texts of 98 articles were examined in further detail and 22 studies were included in the final sample. See Figure 1 for the PRISMA flow diagram.

### Data extraction

The following data were extracted from papers that met the inclusion criteria: (1) number of study participants; (2) mode of sleep assessment (i.e., actigraphy, sleep diary, etc.), (3) sleep variables (i.e., number of awakenings, longest sleep period, etc.), (4) age at time of sleep assessment, (5) duration of sleep assessment, (6) type of cognitive and/or psychomotor assessment, (7) age at time of cognitive and/or psychomotor assessment, (8) study outcomes comparing sleep variables and cognitive and/or psychomotor variables, and (9) confounding variables (e.g., socioeconomic status, maternal age at conception, breastfeeding status, etc.). The extracted data were organized and grouped as a function of infant age at the time of the sleep assessment. A list of cognitive and psychomotor measures employed in the included studies can be found in Table 1.

**Table 1.** List of Cognitive and Psychomotor Measures

Cognitive/psychomotor measure	Abbreviation
Ages and Stages Questionnaire [39, 40]	ASQ
Bayley Scales of Infant Development [41–43] (Mental Development Index) (Psychomotor Development Index)	BSID (MDI) (PDI)
Developmental Profile-3 [44]	DP3
Fagan Test of Infant Intelligence [45]	FTII
Gross Motor Checklist [46]	GMCL
MacArthur-Bates Communicative Developmental Inventory [47]	MCDI-SF
Reynell Developmental Language Scales [48]	RDLS
Wechsler Preschool and Primary Scale of Intelligence [49]	WPPSI
Willatts Infant Planning Test [50]	WIPT

### Secondary classification of sleep variables

In an attempt to further document the associations between specific sleep variables and developmental outcomes, a secondary classification was performed. Therefore, 101 associations were extracted from the 22 included studies and classified into 3 categories: (1) significant association (SA), (2) nonsignificant association (NS), or (3) mixed results (MRs). MRs were defined as follows: an association was significant at one timepoint but nonsignificant at an additional timepoint; at the same timepoint, certain outcome variables were significant, but others were nonsignificant; or an outcome variable was significant at one timepoint and significant at an additional timepoint, but with opposite directionality (positive vs negative associations).

### Quality assessment

The methodological quality of the included studies was assessed using the National Institutes of Health Quality Assessment Tool for Observational Cohort and Cross-Sectional Studies [51]. This tool contains 14 items (Yes, No, and Not Applicable options) related to the sampling procedure, sample-sized justification (i.e., power analysis), study design, psychometric properties, and the presence of covariates used in the analyses. As per the tool’s instruction, each study was assigned a rating of *good*, *fair*, or *poor*. The quality assessment was conducted independently by two researchers (B.B. and C.L.) after which discrepancies were discussed and ratings were combined. A third researcher was available in the event that a consensus could not be reached.

### Power estimates

Post hoc power analyses were conducted to assess the adequacy of sample sizes in the included studies using G*Power Version 3.1.9.6 [52]. Two relevant meta-analyses [9, 10] investigating the associations between sleep and cognitive outcomes in children aged 5–13 years old reported mean average effect sizes of *r* = 0.08 and *r* = 0.06. As a result, conventional cutoffs corresponding with small effect sizes [53] (Pearson’s *r* = 0.1, Cohen’s *d* = 0.3, Cohen’s *f*^2^ = 0.02) were used to mitigate the risk of calculation errors. A significance level of α = .05 was used for power calculation estimates.

## Results

Table 2 provides a detailed summary of the papers selected for inclusion and highlights associations found between infant sleep–wake patterns and developmental outcomes. Six studies (27.3%) used objective measures to assess sleep, 11 studies (50%) used subjective measures, and the remaining 5 studies (22.7%) employed a combination of both. The results have been summarized below and grouped as a function of infant age at the time of the sleep assessment: (1) First 48 hours of life (*n* = 2), (2) 4–11 months (*n* = 8), (3) 12 months or greater (*n* = 2), and (4) longitudinal studies using several timepoints (*n* = 10). Studies in which infants’ age overlapped with these groups were assigned to the group of best fit. Studies were additionally organized into subcategories based on whether they used subjective or objective measures or a combination of both.

**Table 2.** Extracted Data From Included Studies

Author (year)	Sample size and quality appraisal	Sleep assessment (duration) and timing	Sleep variables	Cognitive and/or psychomotor assessment	Outcome(s)
First 48 hours of life (*n* = 2)					
Freudigman et al. (1993) [54]	*N* = 36 (20 f, 16 m) Quality appraisal: fair	MMS (24 hours) Age: postnatal days 1 and 2	AS %, QS %, active-quiet transitional sleep %, sleep–wake transition %, wake %, AS bout length, QS bout length, mean sleep period, longest sleep period, arousals in QS	BSID (MDI and PDI) Age: 6 months	*Mixed results* Significant: Day 1: higher sleep–wake transition percentage and shorter mean sleep periods associated with higher cognitive and psychomotor scores Higher longest sleep period and more arousals in QS associated with lower cognitive scores Day 2: longer QS bout length and greater amount of QS were both associated with lower cognitive scores Nonsignificant: Quantity of AS, active-quiet transitional sleep, wake time, and AS bout length not associated with cognitive or motor scores Covariates: sex, mode of delivery (vaginal cesarean section)
Judge et al. (2015) [55]	*N* = 27 Quality appraisal: good	MMS (24 hours) Age: postnatal days 1 and 2	AS, QS, sleep–wake transition, wakefulness, arousals in QS, arousals in AS, mean bout length of QS, mean bout length of AS, mean sleep period, longest sleep period, AS to QS ratio	WIPT (problem-solving) Age: 9 months FTII (facial recognition) Age: 6 and 9 months	*Mixed results* Significant: Higher sleep–wake transition and shorter mean AS bout length on days 1 and 2 were associated with poorer facial recognition memory at 6 months Higher sleep–wake transition (day 2) and frequency of arousals in AS (day 1) were associated with lower problem-solving abilities at 9 months Longer mean sleep period on day 2 was associated with better facial recognition at 6 months Nonsignificant: Sleep–wake transition and number of arousals in AS on day 2 were not related to problem-solving No association between sleep and facial recognition at 9 months No association between wakefulness, longest sleep period, and cognitive outcomes Covariates: none
Five to 11 months (*n* = 8)					
Sun et al. (2018) [56]	*N* = 590 (269 f, 321 m) Quality appraisal: good	BISQ (7 days) Age: 2–11 months (*M* = 5.23)	Sleep duration, nighttime awakenings	BSID (MDI and PDI) Age: 2–11 months (*M* = 5.23)	*Mixed results* Significant: Infants who awoke twice during the night had higher cognitive scores than those who awoke once and ≥3x times per night, after controlling for birth weight, maternal education, breastfeeding, and bed-sharing practices Nonsignificant: Awakenings were not associated with psychomotor scores Total sleep duration was not associated with cognitive or psychomotor scores No sleep variables associated with psychomotor scores Covariates: infant age and sex, birth weight, maternal education level, bedroom sharing, current feeding pattern.
Dearing et al. (2001) [25]	*N* = 62 (29 f, 33 m) Quality appraisal: fair	Telephone interview (4 days) Age: 7 months	Circadian sleep regulation (via periodogram analysis)	BSID (MDI) Age: 24 months RDLS Age: 36 months	*Significant* Significant: More advanced circadian sleep regulation at 7 months predicted better cognitive outcomes at 24 months and language abilities at 36 months Covariates: sex, temperament, maternal education, maternal sensitivity, and parenting values.
Tham et al. (2019) [57]	*N* = 267 (117 f, 150 m) Quality appraisal: good	BISQ (7 days) Age: 6 months	Sleep latency, total sleep duration	Relational memory: deferred imitation, relational binding (eye tracking) Recognition memory: recognition/novelty preference Attentional orienting: visual expectation Age: 6 months	*Mixed results* Significant: Typical sleepers (10–18 hours/day) were better at imitating an action than short sleepers (<10 hours/day) Longer sleep latency was associated with poorer ability to relate an object to a location Nonsignificant: No associations between duration or frequency of nighttime awakenings, and imitating an action, ability to relate an object to a location, or recognition memory No association between sleep onset latency and deferred imitation Covariates: breastfeeding exposure, napping, maternal anxiety and sensitivity, maternal education
Scher et al. (2000) [58]	*N* = 83 (N/A) Quality appraisal: poor	Sleep questionnaire (duration = N/A) Age: 9 months	Sleep composite score	Object permanence test Age: 9 months	*Significant* Significant: Higher composite sleep problem scores were associated with poorer object permanence ability Covariates: none
Lukowski et al. (2013) [59]	*N* = 21 (N/A) Quality appraisal: good	BISQ (7 days) Age: 10 months	Nighttime sleep duration, frequency of night awakenings, daytime nap duration, % of sleep obtained at night	Elicited imitation task Age: 10 months	*Mixed results* Significant: Shorter duration of daytime napping and higher percentages of sleep occurring at night were associated with poorer immediate imitation Shorter duration of daytime napping and more night awakenings were associated with poorer delayed recall generalization Nonsignificant: Nighttime sleep duration and night awakenings unrelated to encoding Nighttime sleep duration and percentages of sleep occurring at night were unrelated to generalization All sleep variables unrelated to recall memory Covariates: none
Scher (2005a) [60] Same sample as Scher 2005b	*N* = 55 (N/A) Quality appraisal: fair	Actigraphy (3 days) Sleep diary (7 days) Age: 8 months	Night-waking index, schedule index, sleep onset time, sleep duration, % of activity per minutes of sleep, number of transitions from sleep to wake, longest sleep period, sleep efficiency, number of awakenings	Gross Motor Checklist Age: 8 months	*Mixed results* Significant: Infants who had achieved the crawling milestone had higher night-waking index scores, more awakenings, and longer sleep durations than non-crawlers Nonsignificant: No differences for sleep onset time, activity percentage, sleep–wake transition, longest sleep segment, sleep efficiency, or schedule index Covariates: infant sex
Scher (2005b) [61] Same sample as Scher 2005a	*N* = 50 (24 f, 26 m) Quality appraisal: fair	Sleep questionnaire (7 days) Actigraphy (3 days) Age: 10 months	Sleep onset time, sleep duration, % of activity per minutes of sleep, sleep efficiency, number of awakenings, night-waking index, schedule index	BSID Age: 10 months	*Mixed results* Significant: Higher motor activity during sleep, more episodes of night waking, and lower sleep efficiency were associated with lower cognitive scores Nonsignificant: No association between sleep variables and psychomotor scores Schedule index and night-waking index (sleep diary), and sleep onset and duration (actigraphy) were not associated with cognitive scores Covariates: none
Gosse et al. (2022) [62]	*N* = 76 (42 f, 34 m) Quality appraisal: fair	Actigraphy (7 days) BISQ (7 days) Sleep diary (7 days) Age: 4–14 months	Day sleep duration, night sleep duration, night awakenings, wake after sleep onset	ASQ Age: 4–14 months	*Mixed results* Significant: Longer nocturnal sleep duration associated with higher gross motor scores (actigraphy) and communication scores (sleep diary) at younger ages. Opposite association in older infants Longer day sleep duration associated with lower problem-solving scores (actigraphy, BISQ, sleep diary), association became stronger with age Longer day sleep duration associated with lower communication and lower fine motor scores (actigraphy) More night awakenings were associated with lower fine motor score (sleep diary) in younger infants. Opposite relationship found in older infants More wake after sleep onset (sleep diary) was associated with higher communication scores in younger infants. Opposite relationship found in older infants More wake after sleep onset (sleep diary) was associated with lower gross motor scores Nonsignificant: Night sleep duration not associated with problem-solving skills, or fine motor scores Day sleep duration not associated with gross motor scores Night awakenings not associated with communication, gross motor, or problem solving WASO was not associated with problem-solving skills or fine motor scores Covariates: infant age
12 months or greater (*n* = 2)					
Bernier et al. (2013) [63]	*N* = 65 (38 f, 27 m) Quality appraisal: good	Maternal sleep diary (3 days) Age: 12 months	Sleep duration, % of total sleep occurring at night	BSID (MDI) Age: 12 months Spin the pots; delay of gratification; shape stroop; baby stroop Age: 2 years WPPSI-III (Information and Matrix Reasoning subscales) Age: 4 years	*Mixed results* Significant: Higher ratio of nighttime/total sleep duration at 1 year old predicted higher cognitive scores at 4 years after controlling for socioeconomic status and cognitive scores at 1 and 2 years Nonsignificant: Sleep duration was not predictive of cognitive scores Covariates: child sex and birth weight, maternal and paternal age and education, family income
Montgomery-Downs et al. (2006) [64]	*N* = 20 (8 f, 12 m) Quality appraisal: good	Actigraphy (5 days) PSG (1 day) Age: 14 months	Sleep start time, sleep end time, total sleep time, sleep efficiency %, mean sleep bout time, immobile %, mean activity in sleep, movement and fragmentation index	BSID Age: 14 months	*Nonsignificant* No SAs Covariates: none
Longitudinal studies (*n* = 10)					
Liang et al. (2022) [65]	*N* = 182 (89 f, 93 m) Quality appraisal: good	BISQ (7 days) Age: 6 and 12 months	Timing of nocturnal sleep onset, nocturnal sleep duration, daytime sleep duration, sleep onset latency, frequency of awakenings, duration of awakenings	BSID III (PDI) Age: 6 and 12 months	*Mixed results* Significant: Longer daytime sleep duration associated with lower gross motor scores at 6 months Longer daytime sleep duration associated with lower fine motor scores at 12 months Greater frequency and longer duration of awakenings associated with lower gross motor scores at 12 months Nonsignificant: Onset of nocturnal sleep, duration of nocturnal sleep and sleep onset latency not associated with BSID scores Covariates: none
Pecora et al. (2022) [66]	*N* = 156 (75 f, 81 m) Quality appraisal: good	BISQ Age: 4 and 8 months	Night sleep duration, day sleep duration, night awakening number, night awakening time	DP3 (cognitive and communication subscales) MCDI-SF Age: 4 (DP3) and 8 (DP3 and MCDI-SF) months	*Mixed results* Significant: Longer daytime sleep duration at 4 months was associated with higher MCDI-SF scores at 8 months Longer daytime sleep duration at 8 months was associated with higher DP3 cognitive scores Nonsignificant: Night sleep duration, night awakening # or time not associated with DP3 or MCDI-SF scores Covariates: temperament, breastfeeding, physical activity, siblings, gender, maternal education, and pacifier use
Pennestri et al. (2018) [15]	6 months: *N* = 388 (182 f, 206 m); 12 months: *N* = 369 (176 f, 193 m) Quality appraisal: good	SAQM-adapted (2 weeks) Age: 6 and 12 months	Sleeping through the night (6-hour window), sleeping through the night (8-hour window)	BSID Age: 6, 12, and 36 months	*Nonsignificant* No SAs Covariates: infant sex, SES, breastfeeding status, co-sleeping status, total sleep duration
Plancoulaine et al. (2017) [67]	*N* = 194 (N/A) Quality appraisal: good	BISQ (duration = 7 days) Age: 6, 12, and 18 months	Night awakenings, day-to-night sleep ratio, total sleep duration, daytime sleep duration, night sleep duration	WPPSI-III Age: 36 months	*Mixed results* Significant: Answering “Yes” vs “No” to night awakenings at 6 months, and lower day-to-night sleep duration ratio at 12 and 18 months was associated with lower IQ scores Longer day time sleep duration at 18 months was associated with higher IQ scores Nonsignificant: Total sleep duration, night sleep duration Covariates: SES, maternal BMI, maternal smoking status, infant birth order, TV watching
Bernier et al. (2010) [68]	*N* = 60 (36 f, 24 m) Quality appraisal: fair	Maternal sleep diary (3 days) Age: 12 and 18 months	Sleep duration, % of total sleep occurring at night, sleep fragmentation	BSID (MDI) Age: 12 months Hide the pots Age: 18 months Spin the pots; delay of gratification; shape stroop; baby stroop Age: 26 months MCDI Age: 18 and 26 months	*Mixed results* Significant: Higher ratio of night/total sleep at 18 months predicted better working memory at 18 months while controlling for SES and cognitive functioning (MDI) at 12 months Higher ratio of night/total sleep at 12 and 18 months predicted better impulse control at 26 months after controlling for SES, working memory, and cognitive functioning (BSID; MDI) at 12 months Nonsignificant: Ratio of nighttime/total sleep at 12 and 18 months did not predict better working memory, set shifting, or inhibitory control at 26 months Total sleep duration and sleep fragmentation (number of awakenings) were not associated with any developmental outcomes Covariates: child age, sex, number of siblings, maternal and paternal age and education, family income
Minard et al. (1999) [69]	*N* = 58 (26 f, 32 m) Quality appraisal: good	MMS (24 hours) Age: postnatal days 1 and 2; 6 months (2 days)	Cyclicity (significant vs nonsignificant)	BSID (MDI) Age: 6 and 12 months	*Mixed results* Significant: Significant cyclicity on day 1 associated with lower 6-month cognitive scores Significant cyclicity at 6 months associated with higher 12-month cognitive scores Nonsignificant: Cyclicity assessed on day 2 was not associated with cognitive outcomes 6-month cyclicity was not associated with 6-month cognitive outcomes Covariates: birth weight, maternal age
Franco et al. (2019) [70]	*N* = 78 (37 f, 41 m) Quality appraisal: good	PSG (24 hours) Age: postnatal day 1 and 2; 6 months	Sleep duration, sleep efficiency, AS %, QS %, micro-arousals, micro-arousals in AS, micro-arousals in QS	WPPSI-III (FSIQ, VIQ, PIQ, and GLC) Age: 36 months	*Mixed results* Significant: Infants with longer daytime sleep duration and fewer microarousals during the day had lower FSIQ, VIQ, and GLC scores Higher daytime sleep efficiency was associated with lower FSIQ, PIQ, and GLC scores More nighttime micro arousals were associated with lower VIQ and GLC scores Nonsignificant: Night sleep duration, night sleep efficiency, percent AS, percent QS, microarousals during AS, and microarousals during QS unrelated to cognitive outcomes Covariates: maternal age at delivery, tobacco consumption and anxiety score during pregnancy, SES, sex, gestational age, breastfeeding duration
Becker et al. (1981) [71]	Two sub-groups: *N* = 15 (7 f, 8 m) and *N* = 14 (8 f, 6 m) Quality appraisal: fair	In-laboratory observation (2–5 weeks = 7 hours; 3–12 months = 2 hours) Age: 2, 3, 4, 5 weeks, 3, 6, 12 months	REM storms, AS quantity, QS quantity, REM sleep quantity	BSID Age: 12 months	*Mixed results* Significant: In 2 samples, more REM storms at 6 and 12 months were associated with poorer cognitive outcomes Nonsignificant: AS, QS, and total REM at all ages not correlated with cognitive outcomes Covariates: none
Pisch et al. (2019) [72]	*N* = 40 (21 f, 19 m) Quality appraisal: good	Actigraphy and BISQ (7 days) Age: 4, 6, 8, and 10 months	Wake after sleep onset, night sleep duration, night-waking frequency, daytime sleep duration	Eye tracking Age: 4, 6, 8, and 10 months	*Mixed results* Significant: Infants who were awake for less time during the night showed earlier signs of memory maturation Nonsignificant: Night sleep duration, night-waking frequency, daytime sleep duration Covariates: sex, number of siblings, sleeping arrangement, and maternal education
Spruyt et al. (2008) [73]	*N* = 20 (7 f, 13 m) Quality appraisal: good	Actigraphy and sleep diary (3 days) Age: 3, 6, 11, and 12 months	Diurnal sleep, nocturnal sleep, total sleep	BSID Age: 12 months	*Nonsignificant* No SAs Covariates: none

Abbreviations: N/A, not available; m, male; f, female; SES, socioeconomic status; REM, rapid eye movement; BSID, Bayley Scales of Infant Development; MDI, Mental Development Index; PDI, Psychomotor Development Index; FTII, Fagan Test of Infant Intelligence; MMS, Motility Monitoring System; WIPT, Willatts Infant Planning Test; PSG, polysomnography; WPPSI-III, Wechsler Preschool & Primary Scale of Intelligence—Third Edition; FSIQ, full-scale intelligence quotient; VIQ, verbal intelligence quotient; PIQ, performance intelligence quotient; GLC, general language composite score; RDLS, Reynell Developmental Language Scales; BISQ, Brief Infant Sleep Questionnaire; SAQM, Self-Administered Questionnaire for the Mother—Adapted; ASQ, Ages and Stages Questionnaire; DP3, Developmental Profile; MCDI-SF, MacArthur-Bates Communicative Development Inventory—Short Form; MCDI, MacArthur-Bates Communicative Development Inventory; QS, quiet sleep; AS, active sleep; SA, significant association.

### Power calculation estimates

Power analyses were conducted for 19 of 22 studies. There were three [62, 70, 72] for which power analyses could not be performed in G*Power as these studies used linear mixed effects, generalized linear, or multilevel modeling. However, given the sample sizes of these 3 studies and the small effect sizes being used for the calculations, it is likely that power would have fallen below 0.80. Overall, power ranged from 0.063 to 0.876, with only one study [56] meeting the power requirements to detect a small effect size. Due to the low power of the included studies, there exists an increased risk that type II errors were made and thus the following nonsignificant results should be interpreted with caution.

### First 48 hours of life (*n* = 2)

#### Objective measures.

Two studies investigated the association between sleep and development in the first 48 hours of life. Freudigman and Thoman [54] assessed the sleep of infants (*n* = 36; power = 0.089; quality = fair) using the Motility Monitoring System [74, 75] (MMS; a pressure sensitive pad) and found that higher sleep–wake transition percentage and shorter mean sleep periods on the first day of life were associated with better cognitive and psychomotor scores at 6 months (BSID). Moreover, greater longest sleep periods and more arousals in QS were associated with poorer cognitive scores, but not with psychomotor outcomes. While these associations were not significant on the second day, longer QS bout length and greater QS quantity were associated with poorer cognitive scores (day 2).

Another study [55] also assessed infant sleep (*n* = 27; power = 0.078; quality = good) on the first 2 days of life using the MMS and found that higher sleep–wake transition and shorter mean AS bout length on days 1 and 2 were associated with poorer facial recognition (FTII) scores at 6 months, but not at 9 months. Higher sleep–wake transition (day 2), as well as higher frequency of arousals in AS (day 1), was associated with poorer problem-solving scores at 9 months (WIPT). Additionally, longer mean sleep periods on the second day were associated with better facial recognition (FTII) scores at 6 months.

Taken together, these 2 studies show some negative and positive SAs between sleep in the first 2 days of life and early cognitive or psychomotor development, but results seem inconsistent as a function of the day and sleep variable. Specifically, findings concerning the association between the variables sleep–wake transition percentage and mean sleep period with developmental outcomes appear to be contradictory.

### Five to 11 months (*n* = 8)

#### Subjective measures.

In a study [56] conducted in infants (*n* = 590; power = 0.876; quality = good) ranging from 2 to 11 months old, a lower number of nocturnal awakenings (BISQ; Brief Infant Sleep Questionnaire [76]) was associated with better cognitive (MDI) but not psychomotor (PDI) scores, after controlling for birth weight, maternal education, breastfeeding, and bed-sharing practices. Total sleep duration was not associated with cognitive or psychomotor outcomes. In another study [25], less variability in circadian sleep–wake cycles across 4 nights of sleep (*n* = 62; power = 0.148; quality = fair) at 7 months (telephone interviews) was associated with better cognitive ability (MDI) at 24 months and better language development (RDLS) at 36 months, while controlling for sex, temperament, maternal sensitivity, parenting values, and education.

Tham et al. [57] divided (*n* = 267; power = 0.648; quality = good) 6-month-old infants into 2 groups based on parental-report (BISQ) of total 24-hour sleep duration: (1) short sleepers (less than 10 hours each day) and (2) typical sleepers (10–18 hours each day). Typical sleepers performed better than short sleepers (6 months) on a memory task that involved the infant imitating three actions of an experimenter. Longer sleep latency was associated with a poorer ability to relate an object to a location at 6 months. No associations were found between the duration or frequency of nighttime awakenings and developmental outcomes. Another study from Scher et al. [58] using the Sleep Questionnaire [77] showed that higher sleep problem scores (*n* = 83; power = 0.147; quality = poor) at 9 months (composite sleep problem score) were associated with poorer object permanence ability (9 months). Lukowski and Milojevich [59] used the BISQ to assess sleep (*n* = 21; power = 0.072; quality = good) at 10 months of age and found that shorter duration of daytime napping and higher percentages of sleep occurring at night were associated with poorer immediate recall (imitation task). Furthermore, shorter duration of daytime napping and more night awakenings were associated with poorer generalization ability, while nighttime sleep duration was not associated with any developmental outcome.

#### Objective and subjective measures.

Other studies assessed the association between sleep and development between 5 and 11 months using both objective and subjective sleep measures. Scher [60] assessed infant sleep (*n* = 59; power = 0.084; quality = fair) using actigraphy and the Sleep Questionnaire [78] at 8 months of age and found that infants who had greater nocturnal sleep fragmentation (composite score of number of interrupted nights, number of awakenings per night, and average time spent awake) and longer sleep duration had better gross motor ability (GMCL). More nocturnal awakenings were also associated with better gross motor ability, while the longest continuous sleep period and sleep efficiency were not associated with gross motor ability. In another study using actigraphy and a sleep questionnaire (*n* = 50; power = 0.106; quality = fair), which appears to use the same sample, Scher [61] found that more motor activity during sleep, more episodes of night waking, and lower sleep efficiency were associated with poorer mental ability (MDI) in infants aged 10 months. Nocturnal sleep duration was not associated with developmental outcomes and no SAs were found between sleep variables and psychomotor ability (PDI). Gossé et al. [62] (*n* = 76; power could not be determined; quality = fair) assessed sleep (4–14 months of age) using actigraphy, the BISQ, and a sleep diary and observed that longer nocturnal sleep duration was associated with better gross motor (actigraphy) and communication ability (sleep diary; ASQ) in younger infants, while the opposite was found in older infants. Longer daytime sleep duration throughout the age range was associated with poorer problem-solving (actigraphy, BISQ, and sleep diary), communication (actigraphy), and fine motor abilities (actigraphy). Increased frequency of night awakenings (sleep diary) was associated with poorer fine motor ability in younger infants and better ability in older infants. Lastly, higher wake after sleep onset (WASO) was associated with better communication and poorer gross motor abilities at all ages (sleep diary).

In summary, the results from the above studies investigating sleep between 5 and 11 months old show some negative and positive SAs between sleep patterns and developmental outcomes. Interestingly, while some studies showed that poorer sleep was associated with better development, others showed the contrary. Again, an important number of associations were not significant, and others were inconsistent or even contradictory.

### 12 months or greater (*n* = 2)

#### Subjective measures.

Bernier et al. [63] (*n* = 65; power = 0.202; quality = good) employed sleep diaries to assess the sleep of infants at 12 months and found that while not associated with general cognitive ability (MDI), higher ratios of night-to-total sleep were associated with better executive functioning and reasoning abilities (WPPSI) after controlling for socioeconomic status (SES) and previous developmental outcomes. Total sleep duration was not associated with any developmental outcome.

#### Objective measures.

In the same age range, Montgomery-Downs and Gozal [64] (*n* = 20; power = 0.070; quality = good) did not find any association between any sleep variables measured with actigraphy at 14 months (total sleep time, sleep efficiency, mean sleep bout time, immobile percentage, mean activity in sleep, movement and fragmentation index) and infant mental (MDI) or psychomotor ability (PDI).

In this age and study design category, only the ratio of nighttime sleep to total sleep was associated with better cognitive development.

### Longitudinal studies (*n* = 10)

#### Subjective measures.

Sleep was assessed at multiple timepoints in 10 studies. Liang et al. [65] (*n* = 182; power = 0.270; quality = good) assessed the sleep of infants at 6 and 12 months using the BISQ and found that longer daytime sleep duration was associated with poorer gross motor (6 months) and fine motor ability (12 months; PDI). Additionally, greater frequency and longer duration of awakenings were associated with poorer gross motor ability at 12 months. Onset of nocturnal sleep, duration of nocturnal sleep, and sleep onset latency were not associated with motor ability (PDI). Another study (*n* = 156; power = 0.222; quality = good) used the BISQ to assess sleep at 4 and 8 months of age and found that higher quantities of daytime sleep at 4 and 8 months were associated with better language ability (MCDI-SF) and developmental scores (DP3), respectively, while adjusting for temperament, breastfeeding, physical activity, siblings, gender, maternal education, and pacifier use [66]. Night sleep duration, number of night awakenings, and duration of night awakenings were not associated with language or developmental outcomes.

Pennestri et al. [15] assessed sleep consolidation of infants at 6 (*n* = 388; power = 0.490; quality = good) and 12 months (n = 369; power = 0.460; quality = good) using the question, “During the night, how many consecutive hours does your child sleep without waking up?”. Sleeping through the night was operationalized as 6 or 8 hours of consecutive sleep and was not associated with cognitive or psychomotor functioning at 6, 12, and 36 months. Another multi-timepoint study [67] (*n* = 194; power = 0.254; quality = good) assessed sleep (BISQ) at 6, 12, and 18 months of age and found that more frequent night awakenings were associated with poorer developmental scores (WPPSI) at 6 months while controlling for potential confounding variables (e.g., SES, maternal BMI, and smoking status). However, at 12 and 18 months this association ceased to be significant. Moreover, lower day-to-nighttime sleep ratio at 12 and 18 months, but not 6 months, was also associated with poorer developmental scores (WPPSI). Longer daytime sleep duration at 18 months was associated with better development scores. Lastly, total sleep and nighttime sleep duration were not associated with cognitive functioning.

Bernier et al. [68] (*n* = 60; power = 0.145; quality = fair) assessed sleep using sleep diaries and found that infants with higher ratios of night-to-total sleep at 12 and 18 months had better impulse control, controlling for SES, working memory, and cognitive functioning (MDI). Higher ratios of night-to-total sleep at 18 months were also associated with better working memory and cognitive ability (MDI) scores. Total sleep duration and sleep fragmentation (number of awakenings) were not associated with any developmental outcome.

#### Objective measures.

Minard et al. [69] (*n* = 58; power = 0.120; quality = good) used the MMS to assess sleep on the first and second postnatal days, as well as at 6 months of age and divided infants into 2 groups (significant periodicity and nonsignificant periodicity) based on a cyclicity score (defined as periodicity and predictability of QS bouts). Infants in the significant versus nonsignificant groups were infants whose sleep cycles were deemed to be regular verses irregular, respectively. Infants with more developed cyclicity at 6 months were found to have better cognitive abilities (MDI) than the nonsignificant group (second day was not significant). Another study [70] (*n* = 78; power could not be determined; quality = good) using polysomnography on the first and second postnatal days and at 6 months found that infants with more daytime sleep, higher daytime sleep efficiency, fewer microarousal during the day, and more arousals at night had poorer cognitive ability (WPPSI) at 36 months. Night sleep duration, night sleep efficiency, and microarousals were unrelated to cognitive outcomes.

Becker and Thoman [71] (group 1: *n* = 15; power = 0.064; group 2: *n* = 14; power = 0.063; quality = fair) assessed infant sleep (2, 3, 4, and 5 weeks; 3, 6, and 12 months) and found that a greater number of REM storms (short intense bursts including eye movement during AS) at 6 and 12 months were associated with poorer cognitive ability (BSID) at 12 months. The amounts of AS and QS were not related to cognitive ability at any timepoint.

#### Objective and subjective measures.

Pisch et al. [72] (*n* = 40; power could not be determined; quality = good) used actigraphy and the BISQ to assess infant sleep at 4, 6, 8, and 10 months of age and found that infants who spent less time awake during the night displayed earlier signs of memory maturation (working memory task). However, no association was found between average night-waking frequency, nighttime sleep duration, daytime sleep duration, and cognitive outcomes. Another longitudinal study [73] (*n* = 20; power = 0.070; quality = good) measured sleep using a sleep diary and actigraphy at 3, 6, 11, and 12 months of age and found no significant relationships between any sleep variable (nighttime, daytime, and total sleep) and developmental ability assessed at 12 months (BSID).

Overall, the studies investigating infant sleep and developmental outcomes using multiple timepoints found few SAs between sleep and developmental outcomes. Yet, like in previous studies using a single timepoint, these results appear to be inconsistent and vary considerably by sleep variable and developmental outcome. For instance, out of the nine longitudinal studies, six studies measured nocturnal sleep duration and none of these studies found SAs with any developmental outcome. Moreover, contradictory associations were also observed for the same sleep variables.

### Classification of specific sleep variables

To better identify which sleep–wake variables were or were not associated with developmental outcomes a secondary analysis was conducted. A total of 101 associations between sleep and cognitive or psychomotor development were extracted from the 22 included studies and classified as significant, nonsignificant, or MRs (significant at one timepoint but nonsignificant at an additional timepoint; at the same timepoint, certain outcome variables were significant, but others were nonsignificant; or an outcome variable was significant at one timepoint and significant at an additional timepoint, but with opposite directionality [positive vs negative associations]). The vast majority were either nonsignificant (*n* = 54, 53.5%) or mixed (*n* = 40, 39.6%) and only six associations (*n* = 6, 5.9%) were categorized as consistently significant. These six SAs were extracted from four different studies [25, 58, 60, 72]. The power of these 4 studies ranged from 0.058 to 0.097 and the power of one study [72] was not estimated due to employing a multilevel modeling analysis. The quality of two studies [25, 60] were rated as fair, one [58] as poor, and the other [72] as good. The following six sleep–wake variables were associated with better developmental outcomes: more developed circadian sleep regulation, lower sleep problem scores, less time awake after sleep onset, more night awakenings, and poorer sleep continuity.

### Summary of the risk of bias appraisals

Fourteen of the 22 studies (63%) included in this review were rated overall as having good methodological quality, 7 were rated as fair (32%), and 1 was rated as poor (5%). In terms of strengths, most studies (72%) measured infant sleep prior to measuring developmental outcomes, and 86% used valid and clearly defined developmental outcome measures. All studies measured varying levels of sleep using multiple variables such as total sleep duration, number of awakenings, and duration of night awakenings. More than half (59%) of the studies measured infant sleep at more than one timepoint and half of the included studies attempted to control for confounding variables such as SES, maternal age, and breastfeeding status.

Additionally, loss to follow-up was reported as less than 20% in approximately half of the studies (45%) and the dropout rates were either not reported or could not be determined for half of the studies (45%). The remaining 9% of studies had losses higher than 20% at follow-up. Rates of participation of eligible persons were not reported by more than three-quarters of the studies (77%), with four explicitly reporting rates over 50%, and one study reporting a participation rate below 50%. Finally, almost all studies (95%) did not provide a sample size justification (i.e., power analysis).

## Discussion

The aim of this review was to survey and synthesize the existing literature on the association between sleep in infants aged 0–18 months and cognitive and psychomotor development. While the association between sleep and daytime functioning is more established in older populations, the current review suggests that this association is not as clear and more complex in infancy. Among the 22 studies included in this review, only 2 [25, 58] reported exclusively significant results, 3 [15, 64, 73] found no SAs, and the remaining 17 found a mix of significant and nonsignificant results between sleep and cognitive/psychomotor outcomes. Examples of MRs include associations being significant at one timepoint but nonsignificant at an additional timepoint [54, 55, 66, 67, 70, 79]; at the same timepoint, certain outcome variables were significant, but others were nonsignificant [54–57, 59, 61–63, 66, 68, 70, 79]; or an outcome variable was significant at one time point and significant at an additional timepoint [62], but with opposite directionality (positive vs negative associations). The observed lack of clarity may be attributed, in part, to the low power in the included studies, potentially contributing to the nondetection of SAs and increasing the likelihood of type II errors.

While approximately two-thirds of studies (*n* = 14) were rated as having “good” methodological quality, the other one-third of studies were rated as fair (*n* = 7) and poor (*n* = 1) and thus the results from these studies should be interpreted while taking this into account. The majority of studies rated as “good” were found to have MRs (*n* = 11) and the remaining did not find any SAs (*n* = 3). In contrast, as it will be further developed below, the single study [58] rated as “poor” was one of two studies which found exclusively SAs. Studies rated as fair also found mostly MRs (*n* = 6) and one found exclusively significant results.

One (*n* = 83; power = 0.147; quality rating = poor) [58] of the 2 studies reporting exclusively SAs showed that a higher sleep problem composite score at 9 months was associated with poorer object permanence ability. This study only used a single sleep variable (although based on a sleep composite score) as well as a single developmental outcome variable (object permanence). Not including covariates in the analysis mainly contributed to the “poor” rating of this study. The second study (*n* = 62; power = 0.148; quality rating = fair) [25] reporting exclusively SAs demonstrated that more developed circadian sleep regulation at 7 months was associated with better mental and language development. However, it is important to note that this study relied on a single sleep variable (circadian sleep regulation).

In contrast, the three studies [15, 64, 73] that did not find any SAs all employed multiple sleep variables and developmental variables and were rated as having “good” methodological quality. Notably, two [15, 73] out of three studies employed multi-timepoint designs. Among the 18 studies with MRs, 8 [66–72, 79] utilized a multiple timepoint design, while 9 [54–57, 59–63] relied on a single timepoint. Of these 18 studies, 17 utilized multiple sleep and/or developmental variables. The one exception [25], which used a single sleep and developmental variable, employed a multi-timepoint design.

Studies using a single time point or single sleep outcome variable were of course more likely to yield to exclusively significant results without the presence of additional inconsistencies. Thus, it is possible that these findings would have been categorized as mixed or inconsistent results if multiple timepoints and/or outcome variables had been employed. It is also possible that more sleep variables were tested but not reported given the nonsignificant results, although it is not possible to verify this hypothesis.

In order to better identify which sleep variables were associated or not with developmental outcomes, a secondary classification was performed among the 101 total associations included in the 22 studies. Once again, these results should be interpreted cautiously given the low power estimates of all but one included study. Most of these associations were either nonsignificant (*n* = 54, 53.5%) or mixed (*n* = 40, 39.6%) with only six associations (*n* = 6, 5.9%) being categorized as consistently significant. These six SAs were extracted from four [25, 58, 60, 72] studies, one with a “good” quality rating and two being rated as “fair” and one as “poor.” Dearing et al. [25] (*n* = 62; power = 0.148; quality rating = fair) showed that more developed circadian sleep regulation at 7 months was associated with better mental and language development. Scher et al. [58] (*n* = 83; 0.147; quality rating = poor) found that a higher sleep problem composite score at 9 months was associated with poorer object permanence ability. Another article reporting a SA was from Pisch et al. [72] (*n* = 40; power could not be determined; quality rating = good) showing that infants aged 4–10 months who spent less time awake after sleep onset demonstrated better memory maturation. Lastly, while the fourth study showed two other SAs, these were in the opposite direction of what might be expected. Indeed, Scher [60] (*n* = 59; power = 0.084; quality rating = fair) found that infants at 8 months with poorer sleep continuity and more night awakenings had better gross motor outcomes. Specifically, infants who had achieved the milestone of “crawling” were found to have more night awakenings (>5 minutes) than “non-crawlers.” The authors posited that this may have been a result of brief arousals turning into longer episodes due to the infants’ newly developed locomotion skills. It should be noted that this study appeared to use the same sample as another study included in the review and thus the results of this study should be interpreted with this in mind [61].

The high variability in sleep measures, sleep variables, as well as developmental outcome variables clearly limited the ability to compare the association between sleep and development across studies. Indeed, nine different sleep assessment measures using objective or subjective tools were employed across the included studies, ranging from polysomnography to telephone-based interviews. Additionally, studies also varied widely regarding developmental variables with a total of 11 different instruments being used. Harmonizing sleep and developmental measures would be highly beneficial to this field of research and could improve comparisons between studies.

However, despite the observed variability, some specific sleep variables consistently failed to yield significant results. For instance, among 10 studies measuring nocturnal sleep duration, 8 studies [59, 61, 66, 67, 70, 72, 73, 79] found no SA with development outcomes, 1 study [60] found a SA, and 1 study [62] showed MRs. A similar pattern was observed for total sleep duration over 24 hours, where 6 [56, 63, 64, 67, 68, 73] out of 7 studies failed to find a SA with developmental outcomes, and 1 [57] found MRs. Daytime sleep duration exhibited a more variable pattern, with six [59, 62, 66, 67, 70, 79] studies yielding MRs and two [72, 73] studies finding no SAs with developmental outcomes. Lastly, the ratio of night-to-total sleep also displayed variability with three studies [59, 63, 68] reporting MRs.

While a previous review investigating infant sleep and development by Ednick et al. [33] did not utilize categorizations as those employed in the present review and did not exclude preterm births or neurodevelopmental conditions, the authors arrived at similar conclusions. The authors noted inconsistencies such as changes in directionality of relationships depending on the timing of sleep assessment in included studies. They also observed unexpected associations, such as less total sleep being linked to higher developmental scores in some studies. In line with these findings, the present review arrives at a similar conclusion. Despite employing a systematic search strategy and focusing exclusively on typically developing infants born at term, an association between infant sleep–wake patterns and cognitive and/or psychomotor development could not be conclusively established.

There is a greater amount of literature available investigating this association in children over 18 months of age. A systematic review comprised of 19 studies by Short et al. [10] found that shorter sleep duration patterns between 5 and 13 years old were generally associated with poorer cognitive functioning. Additionally, another systematic review [6] of 26 studies also found that longer sleep durations in 2- to 6-year-old children were associated with better cognitive outcomes, although effect sizes were observed to be small. Moreover, similar results were found in a systematic review [9] of 86 studies showing that school-aged children aged 5–12, with longer sleep duration associated with better executive functioning, cognitive tasks performance, and school performance.

Various factors could explain this differing pattern in infancy, in addition to the fact that both sleep and cognitive functioning are still in development during this period of time. The National Sleep Foundation Guidelines [19] publishes sleep guidelines for infants, children, and adults, and recommend that infants (4–12 months) sleep for 12–16 hours (including naps) each day, resulting in a large range of “normal” sleep duration in infancy. However, the majority of the studies included in this review measure sleep duration using continuous variables. Perhaps one way to better evaluate the consequences of suboptimal sleep during infancy on later development would be to target infants who sleep less than these normative ranges using thresholds versus the continuous variables, allowing for these important interindividual differences. Finally, while 14 studies (64%) incorporated meaningful covariates known to be associated with sleep and development, the remaining 8 (36%) studies did not include any covariates, potentially influencing the reported results. A good example is found in the study of Dearing et al. [25] where the addition of covariates (sex, temperament, maternal education, maternal sensitivity, and parenting values) strengthened the relationship between circadian sleep regulation and developmental outcomes.

### Limitations

The findings of the current review should be considered in the context of various limitations. As discussed previously, the main limitation is the consistent underpowered status across almost all included studies, which may have contributed to the notable proportion of nonsignificant results. However, the power calculation should also be nuanced as a function of the specificity of the sleep measure (for example a questionnaire vs actigraphy or polysomnography). Smaller sample sizes are probably less concerning in studies using objective sleep measures than subjective ones. Moreover, assessing sleep with objective sleep measures, such as polysomnography, in infants may also raise concerns related to participant impacts, ethical considerations, and resource intensiveness, therefore limiting the possibility of recruiting larger sample sizes. An objective measure such as actigraphy may be a better choice given its ability to track infant sleep over several days in their home environment.

Despite the utilization of a systematic search strategy, it is conceivable that some eligible studies may have been inadvertently omitted. Additionally, publication bias may have influenced the results due to excluding non-peer-reviewed studies and gray literature. Indeed, one can wonder if the number of NSs could be even higher due to publication bias regarding the publication of significant results. This review also excluded studies which were not published in English or French and those lacking available translations in these languages. The diversity of sleep assessment tools, sleep variables, and timepoints, precluded the feasibility of conducting a meta-analysis. While the majority of included studies demonstrated good methodological quality, it is noteworthy that almost all of them lacked sample size justifications.

## Conclusion

Results from this systematic review showed that most studies assessing the association between infant sleep and psychomotor or cognitive development yielded mixed or inconsistent findings in this specific developmental period. Results from this review should not be interpreted as a conclusion that infant sleep is not important for healthy development. Instead, it underscores the need for the field of pediatric sleep to refine which sleep variables, at what age, and in what context could more accurately predict optimal or suboptimal indices of development. Due to the low power of the majority of included studies, future investigations should consider increasing sample sizes where feasible to account for nondetection of significant findings. Additionally, perhaps looking at persistent poor sleep patterns and considering interindividual variability could also constitute interesting avenues. Overall, clinicians working with families and infants should be aware that as of today, we do not have sufficient data to describe a causal relationship between sleep patterns and development in early infancy. These two developmental processes are in a constant evolution during early development, are highly variable and are also largely influenced by both biological determinants and the environment.

## Supplementary material

Supplementary material is available at *SLEEP* online.

zsae174_suppl_Supplementary_Materials

## Data Availability

No new data were generated or analysed in support of this research.
