# Supplementary material for: The association between infant sleep, cognitive, and psychomotor development: a systematic review
Source: Sleep. 2024 Sep 4;47(11):zsae174. doi: 10.1093/sleep/zsae174 (PMC11543625; doi:10.1093/sleep/zsae174)
Supplement: zsae174_suppl_Supplementary_Materials [file zsae174_suppl_supplementary_materials.docx]

**The Association Between Infant Sleep, Cognitive, and Psychomotor Development: A Systematic Review**

Bryan Butler^1,2^, Rebecca Burdayron^1,2^, Gil Mazor-Goder^1^, Clara Lewis^1^,

Mélanie Vendette^3^, Bassam Khoury^1^, Marie-Hélène Pennestri^1,2^*

^1^ Department of Educational and Counselling Psychology, McGill University, Montréal, QC, Canada

^2^ Hôpital en Santé Mentale Rivière-des-Prairies, CIUSSS-du-Nord-de-l’île-de-Montréal, Montréal, QC, Canada

^3^ Center for Advanced Research in Sleep Medicine, Hôpital du Sacré-Coeur de Montréal, CIUSSS du Nord-de-l’Ile-de-Montréal, Montréal, Québec H4J 1C5, Canada

*Corresponding author. Marie-Hélène Pennestri, McGill University, Department of

Educational and Counselling Psychology, Education Building, Room 614, 3700 McTavish

Street, Montreal, Quebec, Canada, H3A 1Y2, marie-helene.pennestri@mcgill.ca, 1 + 514-398-8079

Appendix A

Supplemental Material

**Search Strategy**

*Medline*

This version of the database was utilized for the search: MEDLINE® and Epub Ahead of Print, In-Process & Other Non-Indexed Citations, Ovid MEDL®(R) Daily 1946 to Present. The following search terms were used: SLEEP (subject heading), limited by age group (All infants).

*PsycINFO*

PsycINFO 1806 to Present. The following search terms were used: SLEEP (subject heading), exploded, and limited by age group (120 neonatal <birth to age 1 mo> or 140 infancy <2 to 23 mo>).

*SCOPUS*

The default version of SCOPUS was used for the purpose of this search. The following terms were used: (TITLE-ABS-KEY (sleep) AND TITLE-ABS-KEY (infant OR baby OR babies OR infants) AND TITLE-ABS-KEY (cogni“* OR "psycho”moto“" OR "psych”moto“" OR "psycho”motor"). Due to the interdisciplinary nature of SCOPUS, the additional search terms ‘cognitive’ and ‘psychomotor’ were added to limit search results.
